# New Energy, Old Lessons: The Evolving Safety Profile of Pulsed Field Ablation

**DOI:** 10.1016/j.jaccas.2026.108763

**Published:** 2026-07-29

**Authors:** Daniel Campos-Villarreal, William H. Sauer

**Affiliations:** Cardiac Arrhythmia Service, Brigham and Women's Hospital and Harvard Medical School, Boston, Massachusetts, USA

**Keywords:** ablation, atrial fibrillation, ventricular tachycardia

**Figure:**
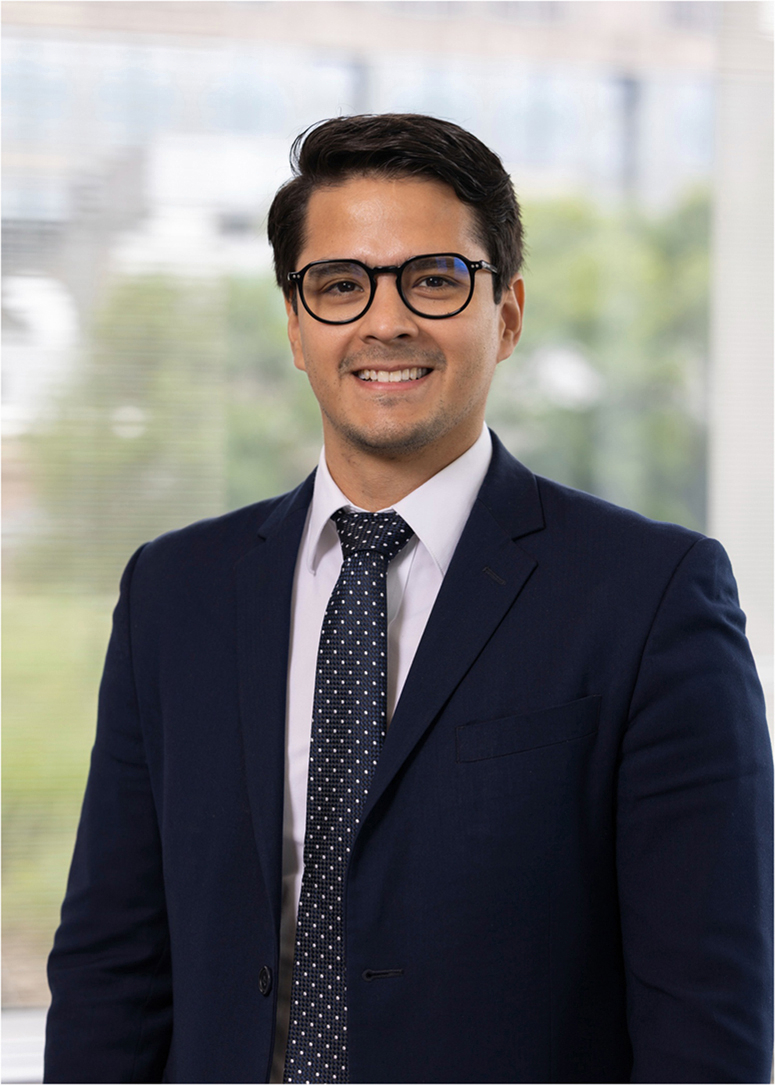

The history of catheter-based cardiac ablation is, in part, a chronicle of unanticipated consequences. Each transformative advance in ablation energy has brought with it a category of adverse effects that neither first-principles physics nor controlled preclinical investigations fully predicted. Radiofrequency ablation, which became the dominant modality more than 3 decades ago, gradually revealed complications that took years of clinical experience to define: the atrioesophageal fistula, phrenic nerve injury from right-sided pulmonary vein isolation, and remote heating of adjacent metallic structures, well before such interactions were systematically studied.^1^ Cryoablation, introduced with enthusiasm for its putative safety advantages, produced phrenic nerve palsy and hazardous catheter adherence, whereas high-intensity focused ultrasound—the basis of the ProRhythm balloon platform—was ultimately abandoned due to unacceptable rates of acoustic energy propagation across tissue interfaces. In every instance, the lesson remains constant: complete characterization of a novel energy modality's safety profile requires broad clinical use, systematic observation, and time.

Pulsed field ablation (PFA) emerged against this backdrop with a mechanistically compelling safety rationale. By exploiting irreversible electroporation rather than thermal injury, PFA preferentially disrupts cardiomyocytes while sparing structures such as the esophagus and the phrenic nerve. However, adoption has been remarkably rapid, and, as is often the case with transformative technology, understanding specific failure modes has struggled to keep pace. The report by Hayasaka et al in this issue of *JACC: Case Reports* is a timely reminder that PFA is now writing its own chapter of unanticipated lessons.^2^

The interaction between ablation energy and metallic implants was not uncharted territory entering the PFA era. Research conducted a decade ago established that radiofrequency ablation in proximity to intracardiac metallic components significantly potentiates local tissue heating due to current density concentration at the metal–tissue interface, with consequent ohmic heating.^1^ The physical principle—that conductive metallic structures distort surrounding electrical fields and concentrate current density—is not energy-source dependent; it applies equally to high-voltage electric pulses. More recently, preclinical data documented visible arcing when PFA was delivered in direct contact with left atrial appendage occlusion devices, while computational modeling has confirmed that conductive structures can locally amplify field intensity even without direct contact.^3^^,^^4^

The present report gives this concern new urgency by demonstrating that hazards can manifest in forms that existing monitoring parameters cannot detect. In cases involving a lattice-tip catheter adjacent to septal occluders, epicardial clips, and transcatheter valves, catheter temperatures reached peak values of 53 to 63 °C, with ΔT of 17 to 32 °C above baseline.^2^

Remarkably, in none of these cases did impedance change appreciably, nor did intracardiac echocardiography show the abnormal bubble formation typically indicative of tissue injury; these 2 routine surrogate markers used to detect energy delivery issues were effectively blind to the hazard. This represents a new qualitative safety concern: not a known risk in a familiar form, but a familiar risk in an unfamiliar and clinically silent form.

This failure is a consequence of contemporary system architectures in which impedance reflects bulk tissue conductivity rather than focal interface conditions. When a small metallic implant concentrates current density and generates localized heating, the resulting impedance perturbation is too focal to register—analogous to trying to detect a single hot spot in a room by measuring average air temperature at the doorway. This is not a device-specific limitation, but an inherent property of these systems. The corollary is consequential: on-catheter temperature sensing, an accompanying feature of dual-energy platforms, provides the only available safety signal. In systems lacking this capability, such heating remains completely invisible.

The findings of Hayasaka et al,^2^ together with emerging reports of esophageal injury near occlusion devices attributed to thermal injury rather than electroporation,^5^ intraprocedural microbubble formation,^6^ and transient silent cerebral lesions,^7^ warrant systematic investigation and remind us that PFA's biological footprint remains incompletely characterized. None of these were confidently predicted from preclinical data; each has become apparent through broad clinical use and careful observation. Mechanisms should inform confidence, not replace vigilance.

Until prospective data define these risks more precisely, direct contact between PFA catheters and metallic implants must be avoided. Intracardiac echocardiography should serve to assess catheter proximity to implants and to monitor for abnormal bubble formation, recognizing that the absence of bubbles does not exclude significant focal heating. For operators using nonsensing focal temperature platforms, these observations should heighten awareness that standard warning metrics may be insufficient in this setting.

PFA represents a genuine advance in the ablation armamentarium, and its tissue-selective mechanism justifies the evidence-based clinical enthusiasm driving its adoption. But history counsels a familiar discipline: no energy modality teaches all its lessons in controlled trials. The field's obligation is to approach these remaining unanticipated interactions with systematic rigor and epistemic integrity.

## Funding Support and Author Disclosures

Dr Sauer has received research funding support and consulting fees from Biosense-Webster. Dr Campos-Villarreal has reported that he has no relationships relevant to the contents of this paper to disclose.
